# Response to Letter to the Editor from Edmundo Avila-Hipolito: “Long-Term Effects of Radioiodine in Toxic Multinodular Goiter: Thyroid Volume, Function, and Autoimmunity”

**DOI:** 10.1210/clinem/dgaa560

**Published:** 2020-08-19

**Authors:** Tania Pilli, Catarina Roque, Francisco Sousa Santos, Gilda Dalmazio, Maria Grazia Castagna, Furio Pacini

**Affiliations:** 1 Section of Endocrinology, Department of Medical, Surgical and Neurological Sciences, University of Siena, Siena, Italy; 2 Section of Endocrinology, Diabetes and Metabolism, Department of Internal Medicine, Egas Moniz Hospital–Occidental Hospital Centre in Lisbon, Lisbon, Portugal

**Keywords:** toxic multinodular goiter, hyperthyroidism, radioiodine, fixed activity, 15 mCi, volume reduction

In response to Avila-Hipolito et al (1), in our study the majority of patients showed subclinical hyperthyroidism (SHyper, 88.2%). Of these, 82% had SHyper type 1 (thyrotropin [TSH] 0.1-0.39 mcU/mL) and the remaining SHyper type 2 (TSH < 0.1 mcU/mL), with a mean age of 66.2 ± 8.3 and 69.0 ± 8.3 years, respectively. According to the American and European guidelines for treatment of SHyper, radioiodine (RAI) is recommended in patients older than 65 years with grade 2 SHyper (strong recommendation) and in grade 1 SHyper, particularly in the presence of comorbidities (eg, heart disease), which were present in the majority of patients in our study (2-5). We agree that the latter is a weak recommendation; however, RAI may be indicated in autonomously functioning nodules also to reduce thyroid volume and to alleviate compressive symptoms, in case of contraindications to surgery or patient preference (6). Moreover, SHyper associated with a multinodular goiter (MNG) may be stable over the time, but in iodine-deficient areas, such as Italy, an iodine load (eg, administration of iodine-containing medications such as amiodarone or iodinated imaging agents) may favor its progression to overt hyperthyroidism (7). Our study has some limitations, but iodine intake and medical therapy are not likely to be confounding factors because iodine contamination was ruled out (urinary iodine excretion, mean ± SD: 106 ± 107 g/L) and it is known that, beside its excess, body iodine content is not an important determinant of thyroid ablation (8). Regarding the use of antithyroid drugs, before RAI all patients were treated with methimazole for hyperthyroidism control and the drug was withdrawn 20 days earlier, allowing the reduction of TSH at pretreatment levels in the majority of the patients. After radioiodine, according to our clinical practice, patients with persistent/recurrent hyperthyroidism were given methimazole. In these cases when the thyroid function was stable at a low dose of methimazole (2.5-5 mg/day), the antithyroid drug was stopped and thyroid function was reevaluated to assess the possible restoration of the euthyroidism. Finally, although the number of patients with available data showed the marked decrease over the time, starting from the fourth year after RAI the mean reduction of thyroid volume, compared to the baseline, did not change significantly, suggesting that the greatest reduction of the gland size can be observed at that time.

In conclusion, although prospective studies with a larger cohort of patients are desirable to confirm our data, we think that our findings may support the use of a low, fixed dose of RAI for toxic MNG with a good outcome in terms of hyperthyroidism cure and thyroid volume reduction.

## Data Availability

All data generated or analyzed during this study are included in this published article or in the data repositories listed in References.

## Abbreviations

SHyper: subclinical hyperthyroidism
MNG: multinodular goiter
RAI: radioiodine
TSH: thyrotropin

## Additional Information

***Disclosure Summary:*** The authors have nothing to disclose.
